# Color stability of an artificially aged nanofilled composite resin post-cured with different techniques

**DOI:** 10.34172/joddd.2021.010

**Published:** 2021-02-13

**Authors:** Lais Sampaio Souza, Tais Rocha Donato, Gabriela Alves Cerqueira, Andrea Nobrega Cavalcanti, Paula Mathias

**Affiliations:** ^1^Private Practitioner, Bahia, Brazil; ^2^Institute of Health Science , Federal University of Bahia, Brazil; ^3^Department of Restorative Dentistry , Faculty of Dentistry, Status University of Campinas, Brazil; ^4^Department of Clinical Dentistry, Faculty of Dentistry, Federal University of Bahia, Brazil

**Keywords:** Color, Composite resin, Post-curing

## Abstract

**Background.** Post-cured composite resins exhibit improvements in physical and mechanical properties due to additional polymerization conversion. However, the post-curing techniques might influence the color stability of composite resin materials. Thus, this study evaluated the color stability of a nanofilled composite resin (Filtek Z350 XT - 3M ESPE) subjected to different post-curing techniques.

**Methods.** Sixty samples (color A2) were randomly allocated to six experimental groups (n=10): G1: photoactivation (P) (control); G2: P + microwave oven with distilled water; G3: P + microwave oven without distilled water; G4: P + conventional oven; G5: P + dry-heat sterilizer; G6: P + steam autoclave. All the groups were stored in distilled water for 60 days and immersed daily in 5 mL of a coffee solution for 3 minutes. The color readings (CIEL*a*b* system) were performed at two different time intervals, initially and after 60 days, in a reflectance spectrophotometer (UV-2600; Shimadzu). The colorimetric readings were performed using the Color Analysis software (CIEL*a*b* system).

**Results.** Group G6 exhibited significantly low values of total color change (ΔE=13.16). The control (ΔE=15.32) and G5 (ΔE=15.49) groups exhibited intermediate values, with no difference between them. In turn, the groups in which the resin was heated in a microwave (G2 ΔE=18.55 and G3 ΔE=19.45) exhibited the most significant color changes (one-way ANOVA and Tukey test, *P* ≤0.05).

**Conclusion.** Steam autoclave post-polymerization increased the color stability of the nanofilled resin subjected to artificial aging and coffee immersion.

## Introduction

Composite resins have been extensively used due to the excellent harmony they provide for natural teeth.^1,2^ Although it is a versatile restorative material, it has some disadvantages, such as color instability, especially when exposed to the oral environment.^3^ The color change of this material is one of the main reasons for the replacement of restorations, especially in anterior teeth.^4,5^

The semi-direct technique, also called direct-indirect by Fahl,^6^ is a restorative technique that uses composite resins for posterior and anterior teeth.^2,6,7^ In this technique, the resin is inserted, settled, shaped, and polymerized in the dental preparation, and later removed. Finishing and polishing are performed extraorally and then cemented in the dental preparation.^6,7^ The procedure allows photoactivation of the material from different directions, ensuring a greater range of light in the material. Moreover, it is more comfortable for the patient due to a reduction in intraoral working time; however, it has a limitation of increased working time by the patient chair.^7^

In the semi-direct technique, apart from photoactivation in different directions,^7^ it is possible to add post-curing techniques with the additional use of light, heat, and pressure, either alone or together, to increase the degree of conversion of composite resins, providing complete polymerization and consequently an improvement in physical and mechanical properties.^3,8^ Thus, the chemical degradation of composite resins that occurs mainly due to the diffusion of molecules and non-reacted monomer ions^8^ could be reduced. However, performing one more laboratory step results in increased working time, and it is crucial to verify the effectiveness of this procedure in the color stability of these restorations.

Thus, a post-cured composite resin might exhibit higher color stability due to higher polymerization conversion. Therefore, the hypothesis tested in this study is that the color stability of the nanofilled composite resin is higher when using thermal and thermal/pressure post-curing techniques, with no difference between them. Thus, the present study aimed to assess the color stability of a nanofilled composite resin subjected to five different post-curing techniques, immersion in coffee solution, and the aging process for 60 days.

## Methods

### Production of samples

Sixty samples were prepared for this study using the Filtek Z350 XT (3M Espe, Sumaré, São Paulo, BR) nanofilled composite resin in A2 shade and enamel translucency (A2E). The composition of the nanofilled composite resin is presented in Table 1.

**Table 1:** Description of filler volume, size of the loaded particles, and components of the organic and inorganic phases of the nanofilled composite resin (Filtek Z350 XT - 3M ESPE)

**Resin Components**	**Composition**
Organic matrix	Bis-GMA, UDMA, TEGDMA, Bis-EMA
Inorganic matrix	Non-aggregated silica and zirconia and aggregated silica/zirconia clusters
The fillers size	0.6-10 microns
The filler volume	63.3%

*Note*. Bis-GMA: Bisphenol-A Glycidyl Methacrylate; UDMA: Dimethacrylate Urethane; TEGDMA: Triethylene Glycol Dimethacrylate; Bis-EMA: Bisphenol Hydroxyethyl Methacrylate - resin monomers that form make up the organic matrix.

Sixty samples were prepared (6 mm in diameter and 1.5 mm in thickness) using a split stainless steel matrix placed on a glass plate. The metal matrix was filled with the only increment of composite resin, using a composite resin spatula (Millennium Titanium - Golgran, São Caetano do Sul, São Paulo, BR) and a polyester strip. A 500-g weight was applied and left for 30 seconds to drain the excess material. After removing the weight, the composite resin was photoactivated through the strip for 20 seconds using a light-emitting diode LED (Valo - Ultradent, South Jordan, Utah, USA) with a light intensity of 1400 mW/cm^2^, always in the same position and for the same time for standardization, ensuring that all the samples received the same intensity of LED light. The sample area on which the light-curing device was positioned and activated was the side facing the matrix hole, which was 6 mm in diameter. Next, the samples were identified and stored individually in dark receivers, free of light, at 37°C for 24 hours. After this period, the samples were planed and polished in a metallographic polisher (Arotec - Aropol.vv model - 50-600RPM, Cotia, São Paulo, BR) with sandpapers of 600, 1200, and 2000 grits, under constant water irrigation. The samples were subjected individually to baths in an ultrasonic tank (CBU-100/1L, PLANATC, Tatuapé, São Paulo, BR) with distilled water at 25°C for 2 minutes after polishing with each file. The samples were randomly assigned to six experimental groups (n = 10) according to the post-curing techniques used (Table 2).

**Table 2:** Experimental groups (n=10) divided according to the post-cured techniques used for the nanofilled composite resin tested

**Experimental groups**	**Post-polymerization techniques**	**Manufacturer**
G1 (control group)	Photoactivated resin (P)	
G2	P + microwave oven (480 W for 5 min + 200 mL of distilled water)	Consul Facilite- CMY34ARHNA (São Bernardo do Campo, São Paulo, BR)
G3	P + microwave oven (480 W for 5 min)	
G4	P + conventional oven (127°C for 15 min)	Fogatti F-450X (Blumenau, Santa Catarina, BR)
G5	P + dry heat sterilizer (127°C for 15 min)	Quimis – Q315M15 (Diadema, São Paulo, BR)
G6	P + steam autoclave (127°C [1.5 kgf/cm^2^] for 15 min)	Vitale Class – 12 L Cristófoli (Campo Mourão, Paraná, BR)

### Aging and staining processes

For the aging process, the samples were immersed individually in 5 mL of distilled water at 37°C for 60 days. For the exposure to the dye solution, the samples were immersed daily in 3 mL of coffee solution (Maratá – LOT: 025M03:34; Itaporanga D’Ajuda, Sergipe, BR) at a temperature between 75°C and 85°C, for 3 minutes once a day. The samples were retrieved from the stove 10 minutes before exposure to coffee. During this time, we used an electric coffee machine where we prepared coffee, with 60 g of coffee in 300 mL of water. A plastic case with 10 compartments was used, in which each compartment received a sample. The amount of coffee was sufficient to immerse the sample (3 mL), and a syringe was used. The protocol was standardized using a digital timer that was used to calculate the time of exposure to the coffee solution for each sample. As soon as the first sample was immersed, we started the digital timer and continued adding coffee into the other compartments. In each compartment, the time the immersion started was recorded. Therefore, when the first sample was retrieved after the pre-determined 3 minutes, the others were retrieved successively, according to the initial time, so that each sample was immersed for 3 minutes. This protocol was repeated in each experimental group. At the end of each coffee immersion process, the samples were washed in distilled water and stored again in distilled water for artificial aging.

### Assessment of color parameters according to the CIEL*a*b system

The initial color parameters were measured in a reflection spectrophotometer (UV - 2600; Shimadzu; Quito, Japan) using UV Probe software, which provided the reflectance spectra of the samples in a visible light spectrum of 380‒780 nm. To proceed with this assessment, the samples were placed on a white background (Barium sulfate) aided by a template that allowed reproducing their positioning. Next, the spectral curves were obtained from the reading of each sample and then transported to the Color Analysis software to assess the color, following the parameters of the CIEL*a*b system (Commission Internationale de L’Eclairage), with standardization of the illuminant D65. The referred system corresponds to a universe of three-dimensional color, in which the axes are identified by L*, a*, and b*. The L* coordinate represents the luminosity of an object and is quantified on a scale from zero (pure black) to 100 (pure white). The a* and b* coordinates represent the chromatic characteristics of the object along the green-red and yellow-blue axes, respectively. They come close to zero for the neutral colors (white, grey) and increase their magnitude to more saturated or intense colors. The color was analyzed twice for all the groups: (1) initial and (2) after 60 days of aging and coffee exposure. The parameters L* (luminosity), a* (green-red variation), and b* (blue-yellow variation) were analyzed separately, and the respective values were used to calculate the total color variation (ΔE), applying the formula: ΔE=√(L-L0)^2^ + (a-a0)^2^ + (b-b0)^2^. The values obtained in the color analyses were used to calculate the total color variation ΔE, which corresponds to the variation of the initial and final colors after 60 days.

### Statistical analysis

The power calculation was performed after the pilot study by the BioStat 5.0 statistical program. The analysis indicated that with eight individuals, the study would have >80% power to detect a difference of 3 MPA between the six groups.

Initially, an exploratory analysis of the data was performed to verify the homogeneity of the variances and determine whether the experimental errors presented normal distribution (analysis of variance parameters). The inferential statistical analysis was performed with one-way ANOVA and post hoc Tukey tests for multiple comparisons. The analysis was performed in the SAS statistical software, version 9.1, at a 5% significance level.

## Results

The resin that was photoactivated and post-cured in a steam autoclave presented significantly lower values of color change (ΔE = 13.16) than the other groups (Table 3). There was no significant difference between the control group (ΔE = 15.32) and the resin that was photoactivated and heated in the dry heat sterilizer (ΔE = 15.49). The post-curing performed in the conventional oven (ΔE = 17.89) and the microwave oven (G2 ΔE = 18.55/G3 ΔE = 19.45), regardless of the presence of water, resulted in the highest color changes, with no significant difference between them (Table 3).

**Table 3:** Means and standard deviations of total color variations (∆E) in the experimental groups

**Groups**	**Mean (standard deviation)**	**Tukey test**
Control	15.32 (0.96)	B
Steam autoclave	13.16 (0.79)	C
Dry heat sterilizer	15.49 (1.56)	B
Conventional oven	17.89 (1.33)	A
Microwave oven/water	18.55 (1.88)	A
Microwave oven/without water	19.45 (2.60)	A

Different letters represent statistically significant differences (one-way ANOVA/Tukey test, alpha=5%).

## Discussion

The hypothesis tested in this study that the color stability of the nanofilled composite resin is higher when using thermal or thermal/pressure post-curing techniques, with no difference between them, was partially rejected because the only post-curing technique that increased the color stability was the use of heat and pressure in a steam autoclave after photoactivating the nanofilled composite resin. The other techniques used were not able to increase the color stability; instead, the conventional oven or the microwave oven reduced the color stability of the resin tested significantly, considering the total color change over 60 days.

The resin materials used in direct, semi-direct, and indirect restorations are based on the same formulations but with different polymerization protocols, which might affect the color stability of the material.^9^ This was observed in the present study when different thermal post-curing protocols and those associated with pressure were able to affect the total color change of the composite resin tested differently. The optical properties of composite resins change based on the photopolymerization received, and the extent of change is affected by the characteristics of the material and the wavelength emitted by the polymerizing unit.^4,10,11^ In the present study, both the material and the light-emitting source were standardized so that these variables would not interfere with the results, providing a proper assessment of the post-curing effect. Further studies should be performed to analyze the effect of these variations, especially with new materials and light-emitting sources.

The additional activation methods tested in this study (steam autoclave, dry heat sterilizer, microwave oven, and conventional oven) are used in the semi-direct and indirect techniques to increase the degree of conversion of resins and consequently provide a higher number of cross-links of the organic matrix, with the ability to introduce a composite with greater physical and rigidity stability. This determines a higher microhardness and increases the mechanical properties of the material.^12^ However, in the present study, these methods were not equally effective for the color stability of the nanofilled resin tested.

The color system of the International Laboratory Commission (CIEL*a*b) used in this study to analyze the total color change of the resin tested is widely used and effective to determine the color changes of a resin composite.^13-15^ The value of total color change (ΔE) should investigate materials at least two different times,^16^ as in the present study. Moreover, the standardization of color parameters allows a comparison of the results of other investigations for the color stability of resin materials.^13-16^ Another advantage is that the literature establishes a clinically acceptable value of ΔE≤3.3 for color differences between restorative materials.^17^ In the present study, all the groups exhibited much higher values than the value above. This result might have occurred due to a sum of factors. The first factor might be the material selected because the nanofilled composite resin tested in the present study has shown high staining ability.^3,10^ Another factor that might have contributed to the great color change was the inclusion of daily coffee immersions, with a high staining ability on the composite resin.^10^ Coffee contains many ingredients, among which there is a considerable amount of hydrosoluble dyes and acids.^18^ Hence, coffee staining is considered strong and persistent, although its molecule does not impregnate the material deeply.^10^ The pigmentation mechanism of polymeric materials (polyethylene, polypropylene, polyester, and polyamides) through coffee is explained by the affinity of the coffee pigments for the polymeric phase of the composite resin, leading to the absorption of dyes in the resin material and justifying the pigmentation in all the groups.^9^ In addition, the samples were immersed daily in heated coffee (75 to 85°C), which seems to contribute to greater harm to the structural matrix of the resin.^19^

The present study showed that the additional polymerization performed in a steam autoclave, which provides additional heat and pressure to the resin material previously photoactivated, increased the color stability of the resin tested. This finding might be due to the pressure (1.5 kgf/cm^2^) exerted on the material in the autoclave, considering that heat (127°C) was present in all the other groups in which resin was post-polymerized, without a positive contribution to color stability. Other studies also have shown positive effects for the use of pressure in reducing the number of empty spaces within the polymeric mass, resulting in a lower amount of non-reacted monomers and consequently the increase in mechanical properties.^20,21^

The control group, which only received photoactivation, and the group post-cured in a dry heat sterilizer, exhibited intermediate values of total color variation (∆E), without significant differences between them. Although the heat received by the composite resin in the dry heat sterilizer could increase material hardness^22^ by increasing the reaction among the molecules of monomers that did not react previously with photoactivation,^23,24^ it did not result in higher color stability of the nanofilled composite resin tested in this study. This was probably because the simple application of heat without pressure could not seal the material surface to the chromophore agents of the coffee solution. According to Alkhadim et al,^10^ coffee can cause darkening and yellowing of composite resins, especially when in contact with this solution for more than 8 weeks, and its staining effect is more superficial.

Although the heat applied to the photoactivated resin extended the vibration of methacrylate molecules and free radicals, promoting their approximation and consequent increase in the degree of conversion of the composite, determining better mechanical properties,^22^ the present study did not show benefits in the application of heat alone regarding the color stability of the nanofilled composite tested. On the contrary, the use of additional heat in the nanofilled composite resin as a post-curing process in a conventional oven or a microwave oven significantly increased the superficial staining of the material when immersed in water and coffee for 60 days.

This significant increase in superficial staining of the resin after the application of heat could be explained by the fact that the amines present in the product tested form byproducts during the polymerization process, which tend to cause colorimetric changes under the influence of heat.^25^ In the present study, the heat applied in the ovens (conventional and microwave) increased the staining of the composite resin tested, potentially because of the intrinsic changes of the material at the time of application of the referred post-curing techniques. Moreover, this same material was subjected to posterior sequential exposure to heated coffee. Vitória et al^19^ showed the harmful effects, such as the increase in water sorption and solubility, on the resin material exposed to heated coffee solution. This might justify this greater tendency to accentuated staining, especially in these experimental groups.

Kiran et al^26^ exposed different restorative materials to increased temperatures in an oven to assess the changes in the properties of color and fluorescence. At temperatures >200°C, all the materials tested, including the composite resin, exhibited degradation of their organic components, causing the appearance of gaps and colorimetric changes. In the present study, the average temperature to which the samples were subjected was 127°C, but the findings of color change, especially for the oven groups, might be associated with the same factors, in which a potential change in the structure of the resin matrix predisposed the material to a higher accumulation of pigments from coffee, increasing the significant color changes.

However, other studies affirm that the additional heat exerted on the composite resin previously photopolymerized contributes to a process of annealing it, which might relieve internal stresses by the movement of molecules. Nevertheless, it was found that the humidity during post-curing negatively affects the mechanical properties of the resin.^24,27^ In this study, the presence of humidity did not affect color stability, considering that the comparison of both groups heated in the microwave oven (with and without water) showed no difference in behavior between them.

These additional curing strategies aim to improve the mechanical properties of the material and reduce the effects of polymerization shrinkage observed in the restorations performed using the direct technique.^28^ However, it is required that these post-curing methods do not compromise the optical properties of the materials, especially the color stability of restorations, which is one of the most relevant factors that guarantee the maintenance and longevity of aesthetic restoration in composite resins.

## Conclusion

The color stability of the composite resin stored in water and immersed in coffee changed significantly. The exclusive provision of heat after photoactivating the composite resin could not reduce the total color change of the composite. In turn, the post-curing technique that provided concomitant heat (127°C for 15 minutes) and pressure (1.5 kgf/cm^2^) in a steam autoclave significantly reduced the total color variation of the nanofilled resin material stored in water and immersed in coffee for 60 days.

## Authors’ Contributions

LSS, GAC and TRD contributed in Execution of the study and writing of the article. ANC and PM contibuted in Analysis and interpretation of data. All authors read and approved the final manuscript.

## Acknowledgments

We would like to thank the Institutional Program of Scientific Initiation Scholarships (PIBIC-UFBA) and the Brazilian Council of Technological and Scientific Development (CNPq) for the financial support provided for this study.

## Funding

The funding for this research was offered by the PIBIC-UFBA Program, with the National Council for Scientific and Technological Development (CNPq) as its funding agency.

## Competing Interests

There are no competing financial and non-financial interests.

## Ethics Approval

Not applicable.
